# Letter to editor: Manual acupuncture for the treatment of chronic spontaneous urticaria: a systematic review and meta-analysis

**DOI:** 10.1097/JS9.0000000000003345

**Published:** 2025-09-08

**Authors:** Xiao Han, Min Xia

**Affiliations:** a Department of Rehabilitation Medicine, Ningbo Beilun Third People’s Hospital, Ningbo, China; b Department of Acupuncture Department, Community Health Service Center of Jiaochuan Sub-district, Ningbo, China

*Dear Editor*,

We read with great interest the recently published systematic review and meta-analysis evaluating the efficacy and safety of manual acupuncture (MA) in patients with chronic spontaneous urticaria (CSU)^[1]^. The authors deserve credit for synthesizing available randomized controlled trials (RCTs) and highlighting the potential of MA to alleviate urticaria symptoms and improve patients’ quality of life. Nonetheless, we wish to raise several methodological and interpretative concerns that may affect the robustness and generalizability of the conclusions.

First, although the review included six RCTs with a total of 615 participants, all the evidence originated from studies conducted in China. This geographical concentration raises concerns about external validity, particularly given that cultural acceptance of acupuncture and patient expectations can vary substantially across healthcare systems. Such factors may influence placebo responses, which are known to differ between populations. Broader inclusion of international data from diverse healthcare settings would strengthen generalizability, especially to Western populations.

Second, none of the included trials explicitly reported blinding of participants or outcome assessors, which is a notable limitation when assessing subjective outcomes such as UAS7, DLQI, HAMA, and HAMD^[2]^. The absence of double-blind designs compromises internal validity. Although the authors acknowledged this issue, they did not perform quantitative bias adjustments, such as sensitivity analyses excluding high-risk trials, which weakens the credibility of the pooled estimates. The potential risks of performance and detection bias in such trials should not be underestimated.

Third, several outcome measures exhibited moderate to high heterogeneity (I^2^ = 65–67% for DLQI, PSQI, and HAMD), yet the exploration of heterogeneity sources was limited. While subgroup analyses were performed by intervention duration and risk of bias, these analyses were underpowered and did not adequately address other potential effect modifiers – such as variations in acupuncture protocols (acupoint selection, needle manipulation, treatment frequency) or control group type (sham acupuncture vs. antihistamines). Future studies could employ meta-regression to quantitatively assess these factors.

Although the meta-analysis reported statistically significant improvements in several secondary outcomes (DLQI, PSQI, HAMA, HAMD), the clinical relevance of these changes remains uncertain. For instance, the reported DLQI reduction of 2.16 points may fall short of the minimal clinically important difference for CSU^[3]^. Moreover, substantial heterogeneity (I^2^ > 65%) persisted in multiple comparisons, and residual confounding cannot be excluded despite subgroup analyses. The authors did not perform funnel plot analysis or Egger’s test, citing the small number of included studies (<10), but given the frequent small-study effects observed in acupuncture research, a visual assessment of asymmetry might still provide informative insights^[4]^.

In summary, this systematic review underscores the potential role of acupuncture in CSU. However, future reviews should prioritize high-quality, prospectively registered trials, standardized outcome reporting, and advanced meta-analytic techniques to ensure more reliable conclusions. Well-designed multicenter RCTs with sham acupuncture controls and long-term follow-up will be essential to establish the true clinical value of acupuncture in CSU. Finally, we would like to thank the author for his contributions to the field of acupuncture. This article was created in accordance with the TATIN guidelines^[5]^

## Data Availability

None.
